# Obstructive sleep apnea and 19 gastrointestinal diseases: a Mendelian randomization study

**DOI:** 10.3389/fpsyt.2024.1256116

**Published:** 2024-07-26

**Authors:** Weiheng Yan, Jiayi Zhou, Miaomiao Jiang, Yaru Kong, Han Qin, Yuwei Qi, Shan Wang, Jun Tai

**Affiliations:** ^1^ Department of Otolaryngology, Head and Neck Surgery, Children’s Hospital Capital Institute of Pediatrics, Chinese Academy of Medical Sciences & Peking Union Medical College, Beijing, China; ^2^ National Clinical Research Center for Mental Disorders (Peking University Sixth Hospital), Peking University Sixth Hospital, Peking University Institute of Mental Health, Beijing, China; ^3^ Children’s Hospital Capital Institute of Pediatrics, Chinese Academy of Medical Sciences & Peking Union Medical College, Beijing, China; ^4^ Department of Otolaryngology, Head and Neck Surgery, Children’s Hospital Capital Institute of Pediatrics, Beijing, China; ^5^ Beijing Municipal Key Laboratory of Child Development and Nutriomics, Capital Institute of Pediatrics, Beijing, China

**Keywords:** obstructive sleep apnea, gastrointestinal disease, Mendelian randomization, gastroesophageal reflux, causal relationship

## Abstract

**Background:**

Alterations gastrointestinal diseases (GDs) were reported in individuals with obstructive sleep apnea (OSA), however, the genetic background between OSA and GDs is still unclear.

**Methods:**

This investigation employed Mendelian randomization (MR) analyses to evaluate the causal effect between OSA and 19 types of GDs (gastroesophageal reflux disease (GERD), ulcerative colitis, celiac disease, Crohn’s disease, chronic gastritis, irritable bowel syndrome, primary biliary cholangitis, diverticular disease, gastroduodenal ulcer, acute pancreatitis, non-alcoholic fatty liver disease, primary sclerosing cholangitis, cirrhosis, calculus of bile duct, calculus of gallbladder, pancreatic cancer, gastric cancer, colorectal cancer, and esophageal cancer). The inverse-variance weighted (IVW) method was used to evaluate the main effects model of causality.

**Results:**

This MR study suggests that OSA may play a causal role inflammation-related GDs (GERD, P_IVW_=5.94×10^-9^; gastroduodenal ulcer, P_IVW_=1×10^-4^; chronic gastritis, P_IVW_=0.0214; ulcerative colitis, P_IVW_=0.0296), and gallstones (calculi of the gallbladder, P_IVW_=0.0429; calculi of the bile duct, P_IVW_=0.0068). After accounting for obesity, type 2 diabetes, smoking, and alcohol consumption, the multivariate MR (MVMR) analysis identified that OSA is an independent risk factor for GERD, gastroduodenal ulcer, and calculus of the bile duct. The reverse MVMR analysis showed a causal effect of GERD on OSA. Besides, we did not find that the predisposition to OSA was associated with 4 cancers.

**Conclusion:**

This MR analysis provides compelling evidence of an independent causal relationship between genetically predicted OSA and an elevated risk of inflammation-related GDs. Besides, no causal association was observed between OSA and cancers. Further studies should be carried out to verify our findings.

## 1 Introduction

Obstructive sleep apnea (OSA) is the most prevalent form of sleep-disordered breathing with a global prevalence rate of 9–38% (1, 2). OSA can lead to intermittent hypoxemia, sleep fragmentation, sympathetic hyperactivity, variations in intrathoracic pressure, and disturbances in physiological balance (3). These symptoms greatly decrease one’s quality of life and increase morbidity of other diseases (4, 5).

The quality of sleep and sleep-related breathing disorders constitute significant factors contributing to the development of Gastrointestinal diseases (GDs) (6). Observational cohort studies have consistently indicated a higher prevalence of GDs among individuals with OSA compared to those without, encompassing conditions such as gallstones (7), non-alcoholic fatty liver disease (NAFLD) (8), inflammatory bowel disease (IBD) (9) and gastroesophageal reflux disease (GERD) (10–12). A retrospective study involving 586,377 adults in the USA revealed an independent association between OSA and GERD, accounting for multiple potential confounding variables (13). Continuous positive airway pressure (CPAP) therapy has demonstrated efficacy in alleviating gastroesophageal reflux symptoms and stabilizing NAFLD progression in patients with OSA (14, 15). However, these findings have encountered scrutiny in recent research (16–18). At present, there is limited evidence of associations between OSA and other common gastrointestinal disorders. In summary, the causal link between OSA and GDs remains contentious, with debates centering on potential confounders and reverse causation biases. A comprehensive evaluation of this causal relationship is crucial to unraveling the mechanisms underlying disease co-occurrence and identifying therapeutic strategies that benefit both conditions.

Mendelian randomization (MR) provides a more precise evaluation of causality for exposure–outcome associations by leveraging genetic variations as instrumental variables (IVs) (19–21). By generating genotypes through randomly assigning parental alleles during conception, MR minimizes the possibility of potential residual confounders and reverse causalities that could be influenced by environmental and self-imposed factors (22). MR has been widely used to predict causal pathways in studies examining the effects of OSA on atrial fibrillation (23), interleukin levels (24), and neurodegenerative disease (25). However, the effects of OSA on a broad range of gastrointestinal outcomes have not been investigated.

We performed a two-sample MR analysis and multivariate Mendelian randomization (MVMR) to evaluate whether OSA and common GDs are causally related. In addition, we used the same MR analysis methods in the reverse direction to determine whether GDs increased susceptibility to OSA.

## 2 Methods

### 2.1 Study design and genetic variants

**Figure 1:**
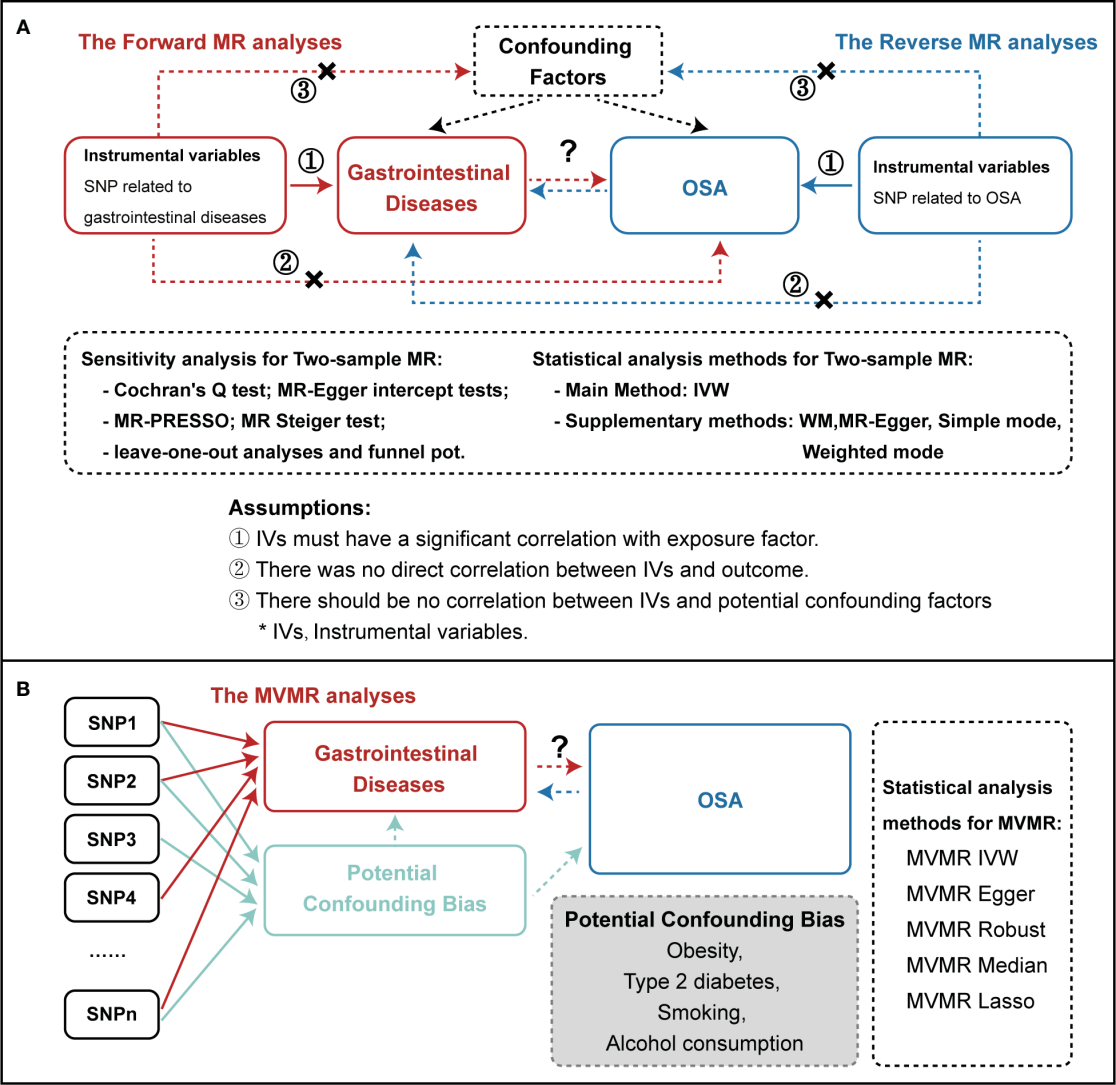
Study flow diagram. **(A)** The flow of Two-Sample MR analysis. **(B)** The flow of MVMR analysis. The dashed lines represent possible pleiotropic or direct causal effects between variables that may violate the MR assumptions. IVW, inverse-variance weighted; WM, weighted median; MR, Mendelian randomization; MR-PRESSO, MR pleiotropy residual sum and outlier; OSA, Obstructive sleep apnea; GDs, gastrointestinal diseases; IVs, instrumental variables; MVMR, Multivariate Mendelian randomization; LASSO, least absolute shrinkage and selection operator.

We performed a bidirectional two-sample MR analysis to explore the causal relationships between OSA and the risk of developing one of 19 GDs. A flowchart of the study design is presented in **Figure 1**. The validity of genetic instruments is based on three critical principles. First, genetic variants employed as IVs should exhibit high confidence in their association with risk factors. Second, the chosen IVs should be independent of confounders that can influence the outcome. Third, IVs should not affect the outcome directly but only through their respective exposure traits (26, 27).

### 2.2 Data sources and IV selection

The analysis used summary-level data from publicly available GWAS projects and consortia to investigate the potential correlation between OSA and 19 GDs. No overlapping data on exposures and outcomes were permitted, minimizing the likelihood of statistical bias. Each GWAS used in the study obtained approval from the relevant ethics committees. **Table 1** provides an overview of the source information for the GWAS data incorporated in this study.

**Table 1:** GWAS summary data sources used in Two-Sample MR analysis and MVMR analysis.

Disease	Source	PMID	Population	Ncase	Ncontrol
Gastroesophageal reflux disease	https://gwas.mrcieu.ac.uk/datasets/ebi-a-GCST90000514/	34187846	European	129080	473524
Celiac disease	https://gwas.mrcieu.ac.uk/datasets/ieu-a-276/	20190752	European	4533	10750
Crohn’s disease	https://gwas.mrcieu.ac.uk/datasets/ukb-a-552/	NA	European	732	336467
Colorectal cancer	https://gwas.mrcieu.ac.uk/datasets/ieu-b-4965/	NA	European	5657	372016
Chronic gastritis	https://gwas.mrcieu.ac.uk/datasets/ukb-d-K11_CHRONGASTR/	NA	European	1790	359404
Irritable bowel syndrome	https://gwas.mrcieu.ac.uk/datasets/ukb-b-17961/	NA	European	1047	461963
Oesophageal cancer	https://gwas.mrcieu.ac.uk/datasets/ieu-b-4960/	NA	European	740	372016
Primary biliary cholangitis	https://gwas.mrcieu.ac.uk/datasets/ebi-a-GCST003129/	26394269	European	2764	10475
Diverticular disease	https://gwas.mrcieu.ac.uk/datasets/ukb-b-10859/	NA	European	8429	454581
Gastroduodenal ulcer	https://gwas.mrcieu.ac.uk/datasets/ukb-d-K11_GASTRODUOULC/	NA	European	3467	357727
Calculus of bile duct	https://gwas.mrcieu.ac.uk/datasets/ukb-b-8268/	NA	European	1706	461304
Ulcerative colitis	https://gwas.mrcieu.ac.uk/datasets/ukb-b-7584/	NA	European	2439	460494
Calculus of gallbladder	https://gwas.mrcieu.ac.uk/datasets/ukb-b-8988/	NA	European	3932	459078
Acute pancreatitis	https://gwas.mrcieu.ac.uk/datasets/ukb-b-19388/	NA	European	1215	461795
Non-alcoholic fatty liver disease	https://www.ebi.ac.uk/gwas/studies/GCST90011885	32298765	European	1483	17781
Primary sclerosing cholangitis	https://gwas.mrcieu.ac.uk/datasets/ieu-a-1112/	27992413	Mixed	2871	12019
Cirrhosis	https://gwas.mrcieu.ac.uk/datasets/bbj-a-105/	NA	East Asian	2184	210269
Pancreatic cancer	https://gwas.mrcieu.ac.uk/datasets/bbj-a-140/	NA	East Asian	442	195745
Gastric cancer	https://gwas.mrcieu.ac.uk/datasets/bbj-a-119/	NA	East Asian	6563	195745
OSA	https://www.finngen.fi/en	NA	European	33423	307648
Obesity	https://www.finngen.fi/en	NA	European	18330	324070
Smoking	https://www.finngen.fi/en	NA	European	2922	133128
Type 2 diabetes	https://www.finngen.fi/en	NA	European	49114	283207
Alcohol abuse	https://www.finngen.fi/en	NA	European	4334	254976

GWAS, Genome Wide Association Study; MR, Mendelian randomization; MVMR, multivariate Mendelian randomization; OSA, Obstructive sleep apnea; GDs, gastrointestinal diseases; Ncase, Number of cases; Ncontrol, Number of control samples.

#### 2.2.1 Data sources of OSA

The statistical GWAS data for OSA were obtained from the FinnGen project (DATA FREEZE 8, https://www.finngen.fi/en), the largest independent GWAS project. The FinnGen data was preferred because it minimized the confounding bias caused by overlapping populations. Therefore, GWAS summary statistics obtained from 33,423 patients with OSA and 307,648 controls were included in this FinnGen study. The criteria for the diagnosis of OSA were an apnea–hypopnea index ≥5/h or respiratory event index ≥5/h (28).

#### 2.2.2 Data sources of GDs

A total of 19 common gastrointestinal diseases were included in this study. The GWAS summary-level data for the relationships between genetic variants and 18 types of GDs were obtained from the Integrative Epidemiology Unit (IEU) open GWAS project (https://gwas.mrcieu.ac.uk/). The GDs included GERD, gastroduodenal ulcer, gastric cancer, Crohn’s disease, ulcerative colitis, colorectal cancer, cirrhosis, primary sclerosing cholangitis, primary biliary cholangitis, chronic gastritis, esophageal cancer, calculus of the gallbladder, calculus of the bile duct, irritable bowel syndrome, celiac disease, diverticular disease, and acute pancreatitis. The summary-level data from the GWAS on NAFLD were obtained from the study by Anstee et al. (29).

#### 2.2.3 Sample overlap between exposure and outcome GWAS summary statistics

The GWAS summary data for OSA utilized in this research was derived from the Finngen Project, an independent GWAS endeavor (28). This guarantees no sample overlap between the OSA GWAS summary statistics and the GDs-related GWAS statistics, thus ensuring the strength of the MR results.

#### 2.2.4 Selection of genetic IVs

For global screening of IVs, genome-wide significance level (p < 1×10^-05^) was used as a threshold for screening IVs significantly associated with exposure factors. This threshold ensures a significant association between IVs and exposure factors while ensuring that as many IVs as possible are obtained for subsequent analysis (30). A minor allele frequency threshold of 0.3 was permitted for palindromic single nucleotide polymorphisms (SNPs). SNPs with low linkage disequilibrium were excluded with a strict r^2^ cutoff of 0.001 and a clumping window greater than 10,000 kb. Detailed information on the SNPs employed as IVs is provided in **Supplementary Table S1**. As shown in **Supplementary Table S1**, the genome-wide significance p value between all IVs included in the analysis and the outcome phenotype was greater than 1×10^-05^. This ensured that there was no direct correlation between IVs and outcome phenotypes. To prevent weak instrument bias, the statistical strength of IVs was evaluated by calculating the F-statistic. When the F-statistic is greater than 10, IV is considered to have good statistical performance (31, 32):


$$\text{F}=\left(\frac{{\text{R}}^{2}}{1-{\text{R}}^{2}}\right)\left(\frac{\text{n}-\text{k}-1}{\text{k}}\right)$$

In this context, R^2^ denotes the fraction of variability in exposure that can be ascribed to genetic variants. The variable *n* denotes the sample size of the exposed phenotype, and *k* represents the total number of IVs included in each MR analysis.

### 2.3 Statistical analyses

All statistical analyses were performed using R version 4.1.1 (R Foundation for Statistical Computing; Vienna, Austria). The two-sample MR analysis was performed utilizing the TwoSampleMR package (version 0.5.6); the MVMR analysis was performed using the MVMR package (version 0.3).

#### 2.3.1 Two-sample MR analysis

The principal method adopted in this research for determining the causal association between OSA and GDs was the IVW approach using a random-effects model. To ensure the reliability of the results and identify potential horizontal pleiotropy, multiple sensitivity analyses were carried out, inclusive of the weighted median, MR-Egger, weighted mode, and simple mode methods. Each of these analyses relies on distinct assumptions necessary for making valid inferences. The IVW method computes the effect as the IVW average of the ratio estimates of SNPs, with first-order weights based on each SNP serving as a valid IV (26, 33). The weighted-median method requires >50% of the chosen genetic instruments to be valid; the weighted mode produces reliable outcomes when the percentage of invalid instruments exceeds 50% (34). The MR-Egger method can yield a valid estimate of causal effects even if all SNPs are invalid instruments because it employs weighted regression without a restricted intercept (35). The weighted mode method is also resilient against invalid or pleiotropic SNPs, taking the IVW empirical density function mode as an effect estimate (36). The reliability of the causal inference conclusions is enhanced when the findings of all the methods are broadly consistent. However, causality inferred from other methods may have greater confidence intervals and standard errors than those derived from IVW (32, 37).

This study’s causal effect estimates of genetically predicted OSA on GDs are expressed as ORs with corresponding 95% CIs. The Bonferroni correction for multiple testing was conducted to correct P values. A P value less than 1.32 × 10^−03^ (0.05/19/2; 2 denotes both forward and reverse MR tests) was considered as strong evidence of a causal association. A value of p<0.05 was considered suggestive of a correlation.

#### 2.3.2 MVMR analysis

Because obesity, T2D, smoking, and alcohol consumption are common risk factors for most GDs and OSA, MVMR analysis was used to estimate the risk effect of OSA on GDs after adjusting for these risk factors (38, 39). By integrating several risk exposures into the MR Analysis concurrently, MVMR elucidates whether each discrete risk exposure has an independent causal effect on the outcome when multiple risk exposures co-exist (40). In this research, OSA was amalgamated with the aforementioned four common risk factors as an exposure variable, and an MVMR model was assembled to assess whether OSA exposure had a direct association with GDs outcomes. Given that collinearity bias may be introduced if excessive exposure variables are incorporated in the MVMR model simultaneously, this study used each common risk factor individually with OSA exposure to construct an MVMR model. MVMR-Robust, MVMR-IVW, and the least absolute shrinkage and selection operator (LASSO) were used to determine the independent effects of OSA on GDs (41). **Table 1** provides an overview of the source information for the risk-factors-related GWAS data used in MVMR analysis incorporated in this study. Reverse MVMR analysis using the same strategy determined the independent effect of GDs exposure on the risk of OSA outcomes.

### 2.4 Pleiotropy and sensitivity analysis

Cochran’s Q-test of the IVW and MR-Egger methods was used to assess heterogeneity. Pleiotropy was evaluated using the intercept of the MR-Egger analysis. The Mendelian randomization pleiotropy residual sum and outlier (MR-PRESSO) approach was used to identify potential outlier SNPs through a global test, repeating the analysis after eliminating the outlying SNPs. This method provides a distortion test that can compare differences before and after removing outliers (37). Funnel plots were used to show the discrete relationships between SNPs and as a complementary basis for removing potential outliers. The Steiger directionality test was utilized to ascertain the causal direction between the assumed exposure and potential outcomes. This test can determine whether the genetic variant used as an IV for the exposure trait is also causally related to the outcome or whether it influences the outcome through the exposure trait (42). Leave-one-out analysis was performed by omitting each SNP to evaluate whether a single SNP influenced or resulted in remarkable MR results (43).

## 3 Results

For this investigation, the globally significant threshold for IVs screening was set at p<1×10^-5^, r^2 =^ 0.001, and a clumping window >10,000 kb. Based on these criteria, 747 SNPs were selected as IVs to evaluate causal relationships linking OSA to all 19 GDs, using OSA as the exposure factor. Unfortunately, when GDs were utilized as exposure factors during reverse MR analysis, the IVs that could be extracted from the genome-wide association study (GWAS) summary data on calculus of the bile duct and acute pancreatitis were insufficient. Consequently, no causal association between these two conditions and OSA was identified. The included IVs are detailed in **Supplementary Table S1**. **Supplementary Table S2** presents the IVs featured in the MR analysis that identified a potential causal link between OSA and GDs.

### 3.1 Causal effect of genetically predicted OSA on GDs

**Figure 2:**
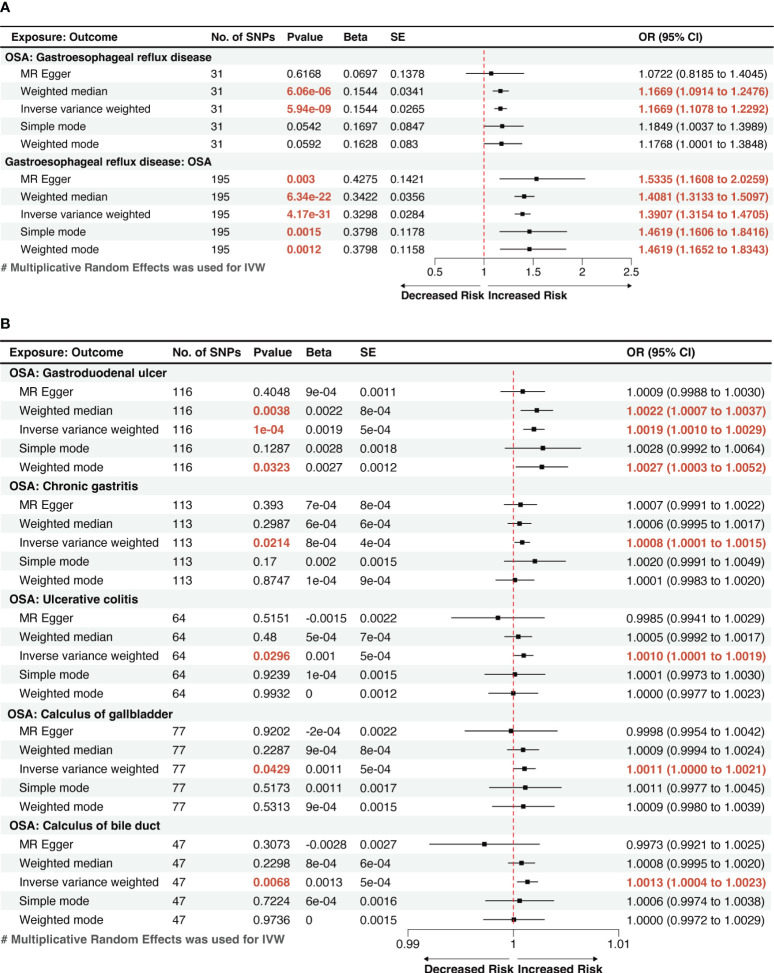
** **A forest plot of the potential causal relationship between OSA and GDs. **(A)** The forest plot of the potential causal relationship between OSA and GERD; **(B)** The forest plot of the potential causal relationship between OSA and other GDs. OSA, Obstructive sleep apnea; GERD, gastroesophageal reflux disease; GDs, gastrointestinal diseases; SNP, single nucleotide polymorphism; OR, odds ratio; SE, standard error; CI, confidence interval; IVW, inverse-variance weighted.

Utilizing the two-sample MR method, with liability to OSA as the exposure and the risk of 19 GDs as outcomes, our analysis found that a genetic inclination towards OSA exhibited an association with 6 types of GDs risk (p < 0.05). According to IVW method, OSA exposure was significantly associated with an increased risk of GERD and gastroduodenal ulcer (p < 1.32 × 10^−03^). Genetically predicted OSA was significantly associated with a 1.1669 greater odds of GERD (95% confidence interval [CI]: 1.1078–1.2292, p=5.94×10^-9^; **Figure 2A**), 1.0019 greater odds of gastroduodenal ulcer (95% CI: 1.0010–1.0029; p=1×10^-4^). And OSA shows a suggestive correlation with 1.0008 greater odds of chronic gastritis (95% CI: 1.0001–1.0015; p=0.0214). The effect estimates from the sensitivity analysis methods (weighted median, MR-Egger, weighted mode, and simple mode methods) were consistent with IVW method.

We also observed suggestive causal associations between genetically predicted OSA with ulcerative colitis (Odds ratio [OR]_IVW_ =1.0010, 95% CI: 1.0001–1.0019, p=0.0296) and gallstones, including calculi of the gallbladder (OR_IVW_ =1.0011, 95% CI: 1.0000–1.0021, p=0.0429) and calculi of the bile duct (OR_IVW_ =1.0013, 95% CI: 1.0004–1.0023, p=0.0068). However, these causal associations were weakened in MR-Egger method, but effect estimates from other sensitivity analysis methods (weighted median, weighted mode, and simple mode methods) were still consistent with IVW method. (**Figure 2B**). The risk of the other 13 types of GDs was not significantly associated with genetically predicted OSA according to the MR analysis.

**Figure 3:**
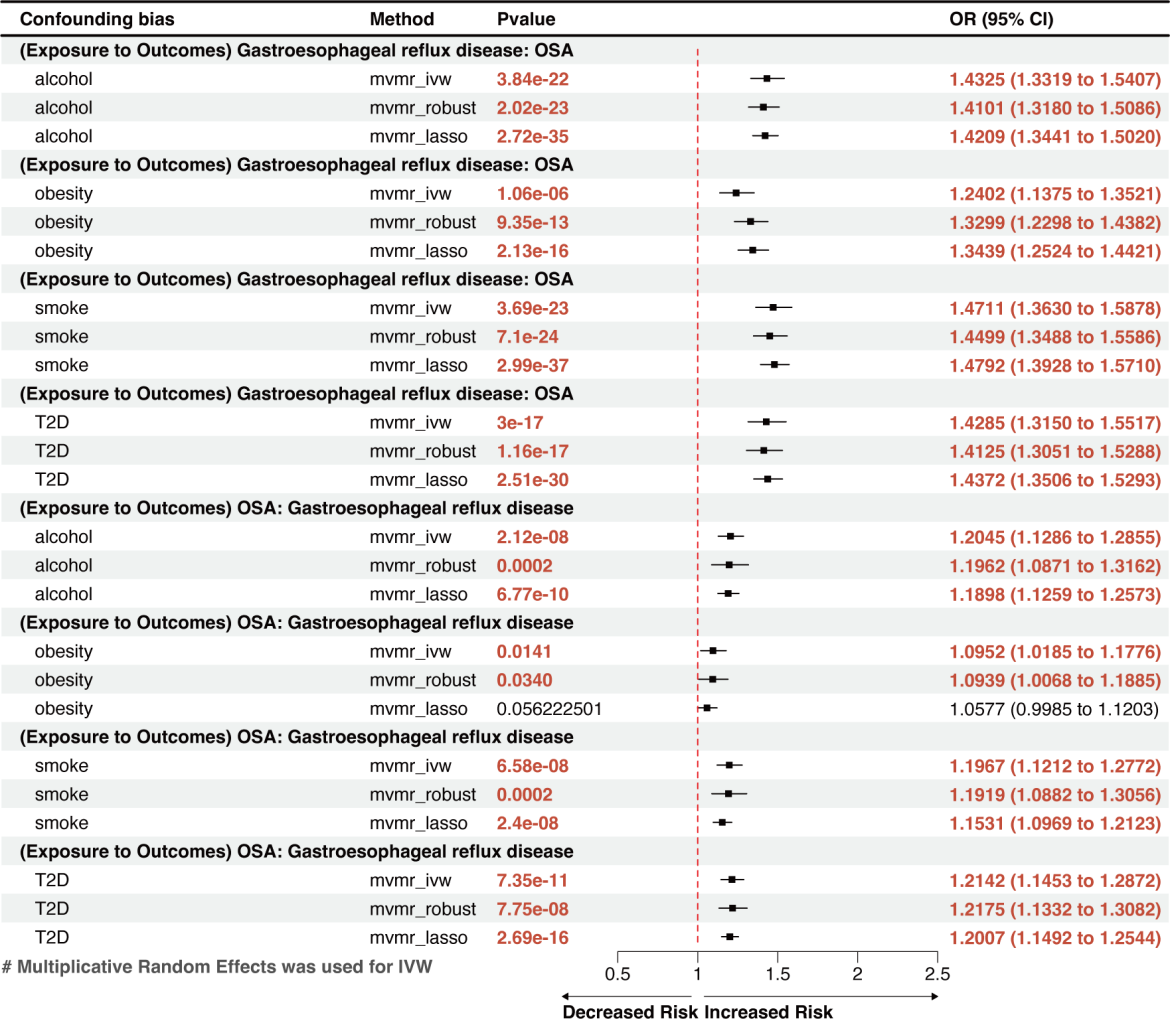
** **A forest plot of the MVMR results between OSA and GERD. OSA, Obstructive sleep apnea; GERD, gastroesophageal reflux disease; OR, odds ratio; CI, confidence interval; IVW, inverse-variance weighted; LASSO, least absolute shrinkage and selection operator; MVMR, multivariate Mendelian randomization.

**Figure 4:**
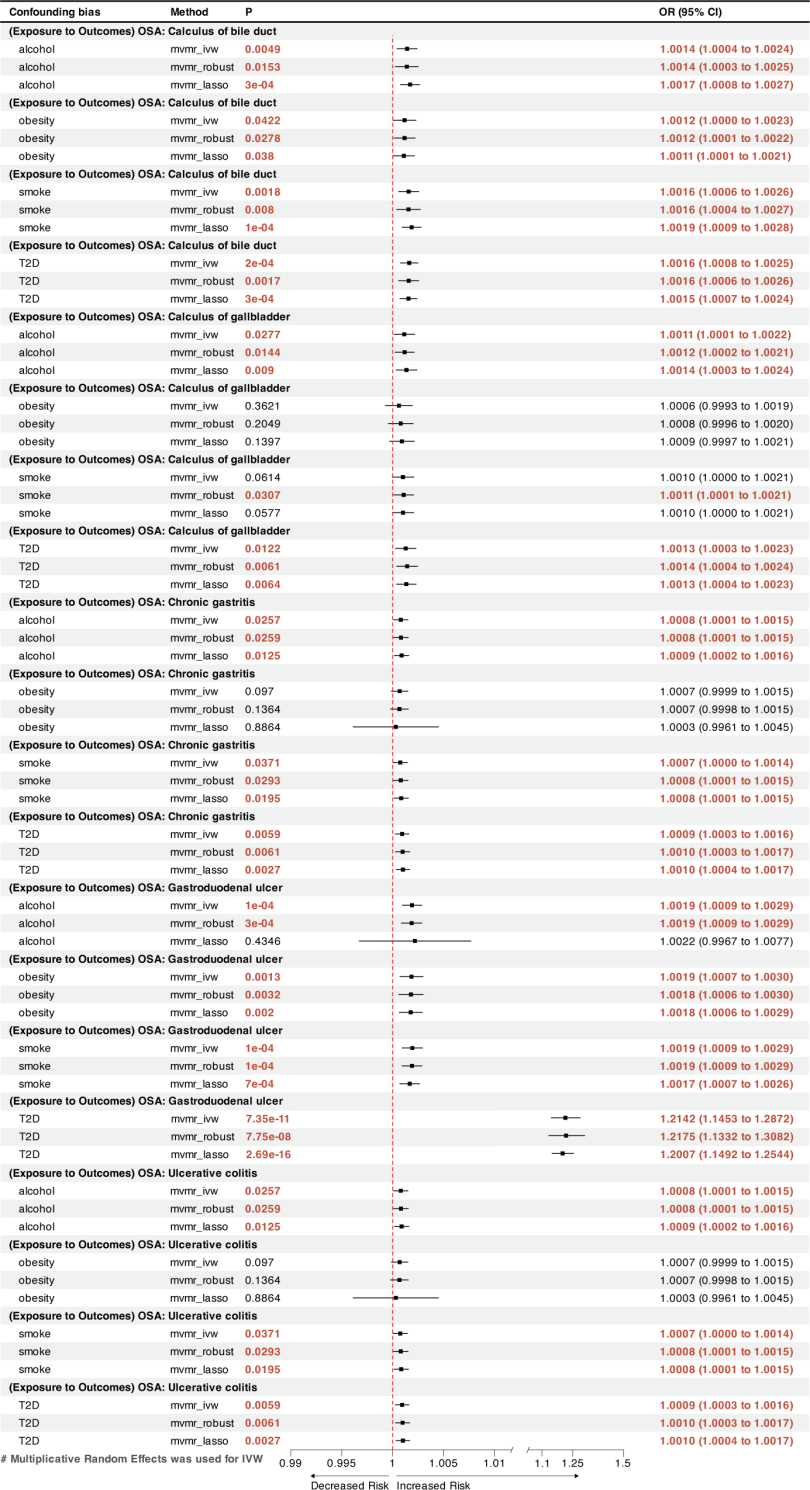
** **A forest plot of the MVMR results between OSA and other GDs. OSA, Obstructive sleep apnea; GDs, gastrointestinal diseases; OR, odds ratio; CI, confidence interval; IVW, inverse-variance weighted; LASSO, least absolute shrinkage and selection operator; MVMR, multivariate Mendelian randomization.

MVMR analysis indicated that OSA was an independent risk factor for GERD, gastroduodenal ulcer, and calculus of bile duct (**Figures 3**, **4**). However, the effects of OSA on chronic gastritis (adjusted OR_IVW_ =1.0007, p=0.0970), ulcerative colitis (adjusted OR_IVW_ =1.0007, p=0.0970), and gallbladder calculus (adjusted OR_IVW_ =1.0019, p=0.3621) were no longer significant in MVMR analysis after considering obesity (**Figure 4**).

### 3.2 Causal effect of genetically predicted GDs on OSA

In the reversed-direction MR study, the potential causal effects of genetically predicted liability for each GD’s exposure on OSA were estimated using the factors found to be statistically significant (p<0.05). Acute pancreatitis and calculus of the bile duct were excluded from the MR analysis because of insufficient IVs (IVs < 3).

The reverse MR analysis showed that GERD had a remarkable causal effect on OSA, demonstrated by an effect estimate of 1.3907 (95% CI: 1.3154–1.4705, p=4.17×10^-31^) for GERD using the IVW method (**Figure 2A**). This result is in line with the outcomes from other methods, including MR-Egger (OR=1.5335, 95% CI: 1.1608–2.0259, p=0.0029) and the weighted-median method (OR=1.4081, 95% CI: 1.3033–1.5097, p=6.34×10^-22^), which showed significant associations with greater confidence intervals. Genetically predicted GERD remained associated with OSA after accounting for obesity, type 2 diabetes (T2D), smoking, and alcohol consumption in the MVMR analysis (**Figure 3**). Other genetically determined GDs were not observed to be significantly associated with OSA risk. The detailed reverse MR results are presented in **Supplementary Table S3**.

### 3.3 Sensitivity analysis

A total of six types of GDs were found to be causally correlated with OSA by Two-sample MR analysis. A total of 331 IVs were used in those MR analysis, corresponding to F- statistic ranging from 137.26 to 1228.67 (F-statistic > 10), which ensured that there was no weak instrument bias in the included IVs (**Supplementary Table S2**). A variety of methods were used to evaluate the dispersion and heterogeneity among IVs. The scatter plots (**Supplementary Figure S1**) and funnel plot (**Supplementary Figure S2**) shows the distribution of IVs in the potential causal relationship between OSA and 6 types of GDs. The leave-one-out analysis showed that single IVs had little effect on the causal association between OSA and GDs (**Supplementary Figures S3**-**S9**).

The Cochran’s Q test was used to identify potential heterogeneity. **Supplementary Table S4** shows Cochran’s Q test results. In the significant causal association between OSA exposure and GDs outcomes, no heterogeneity was found between IVs (P_Cochran’s Q_ > 0.05). When GERD is an exposure factor, the potential causal association with OSA outcome may be influenced by heterogeneity (P_Cochran’s Q_ < 0.05). Therefore, the random-effects IVW method was used in this study to eliminate the bias caused by possible heterogeneity.

The MR-Egger intercept analysis was used for the potential horizontal pleiotropy of MR Analysis between OSA and GDs. On the whole, horizontal pleiotropy was not discovered by MR-Egger intercept analysis. (P > 0.05, **Supplementary Table S5**). MR-PRESSO was used to identify potential outlier IVs and to supplement the evaluation of level pleiotropy. Four potential outliers were detected in the MR-PRESSO analysis of the effect of OSA on GERD. After those IVs removal, horizontal pleiotropy was eliminated. However, in the reverse-MR analysis, possible underlying heterogeneity for the effect of GERD on OSA persisted after eliminating the two SNP outliers (MR-PRESSO Globle Test Pvalue < 0.05, **Supplementary Table S6**). The MR Steiger’s test did not find a potential reverse causal association affecting the robustness of the results (**Table 2**).

**Table 2:** Results of sensitivity analysis of MR Analysis.

Exposure	Outcome	Fstat	Cochrane Q_pvalue	Pleiotropy pvalue	MR-PRESSO pvalue	Stieger test
OSA	GERD	280.91	0.06	0.54	0.10	TRUE
OSA	Gastroduodenal ulcer	323.26	0.35	0.26	0.37	TRUE
OSA	Chronic gastritis	322.16	0.38	0.82	0.38	TRUE
OSA	Ulcerative colitis	315.99	0.63	0.27	0.64	TRUE
OSA	Calculus of gallbladder	318.58	0.45	0.56	0.46	TRUE
OSA	Calculus of bile duct	315.68	0.11	0.13	0.12	TRUE
GERD	OSA	213.01	9.95E-06	0.48	<0.00033	TRUE

MR, Mendelian randomization; OSA, Obstructive sleep apnea; GERD, Gastroesophageal reflux disease; Fstat, F-score used to evaluate statistical power; MR-PRESSO, Mendelian Randomization pleiotropy Residual Sum and Outlier.

## 4 Discussion

This MR analysis is the first to systematically evaluate the cause-and-effect relationship between OSA and digestive disorders. Two-sample MR analysis found a strong correlation between OSA and the incidence of gastroduodenal ulcer and GERD. A nominal causal association was found between OSA and increased risk of calculus of bile duct, calculus of gallbladder, chronic gastritis, and ulcerative colitis. The MVMR analysis found a reciprocal causal link between OSA and GERD. After adjusting for four risk factors (obesity, T2D, smoking, and alcohol consumption) in MVMR analysis, OSA increases the risk of gastroduodenal ulcer and calculus of the bile duct independently.

Numerous epidemiological studies have provided compelling evidence linking several gastrointestinal disorders to sleep disorders such as OSA (6, 10, 44). OSA can induce intermittent hypoxemia, sleep fragmentation, oxidative stress, systemic inflammatory response, and disrupt intestinal flora, potentially contributing to the onset of gastrointestinal diseases. Conversely, nocturnal awakenings, sleep deprivation and sleep fragmentation due to gastrointestinal diseases may reciprocally influence the occurrence of OSA. However, the causal relationship remains unclear due to limitations in temporal sequencing in existing observational studies. To address this gap, we conducted bidirectional MR and MVMR analyses to robustly evaluate the causal connections between OSA and common GDs, providing robust evidence of their causal associations. This findings underscores the importance of early screening for these disorders in OSA-diagnosed patients.

Global population–based studies observed an 8–33% incidence rate of GERD (11). However, individuals with OSA have much greater rates, ranging from 40% to 60% (45, 46). A retrospective study of 586,377 adults in the USA discovered an independent link between OSA and GERD while considering multiple potential confounders (13). However, another multicenter, cross-sectional, observational study conducted in Turkey (n=1104) found that the severity of OSA did not independently predict the occurrence of GERD (17). Recently, an MR study discovered an implied bidirectional relationship between OSA and GERD (47); however, it was unclear whether the results were affected by shared risk factors, particularly obesity. Our findings, which were based on MR techniques that accounted for obesity, smoking, alcohol use, and T2D, indicated a bidirectional, independent causal link between GERD and OSA. This finding can benefit future research on the comorbidity of the two diseases and the optimization of a joint treatment plan.

The supine position itself increases the occurrence of nocturnal GER (48). GER events can be caused by declining intrathoracic negative pressure and elevating abdominal pressure during apneic episodes in OSA patients. This pressure fluctuation also relaxes and opens the lower esophageal sphincter, worsening GERD symptoms (10, 49). Common symptoms of GERD include heartburn or regurgitation, non-cardiac chest pain, chronic cough, and sore throat (50). Nocturnal uncomfortable symptoms can lead to heightened sleep arousal and diminished sleep duration by influencing the interaction of various neural pathways (51). The gastric content of reflux can irritate edema and cause spasms in the upper airway and larynx, thereby contributing to the collapse of the upper airway (10). Additionally, reflux can stimulate the vagus nerve, cause bronchial constriction (52), and affect normal systolic function of the respiratory muscles (53), potentially leading to sleep apnea. The complex pathophysiology underlying their causal relationship warrants further investigation.

The present investigation found previously unrecognized causal links between a genetic predisposition to OSA and a greater incidence of gastroduodenal ulcer and chronic gastritis. An earlier study conducted by Wu et al. found that OSA could affect duodenal morphology via increased oxidative stress and activation of transcription factors, which can lead to the disruption of intestinal tight junctions, culminating in intestinal damage (54). This disruption may lead to the development of gastroduodenal ulcer. Furthermore, multiple cross-sectional studies have shown that shorter sleep durations and poor sleep quality increase the likelihood of gastroduodenal ulcer recurrence (55, 56). The MVMR analysis showed that OSA’s effect on chronic gastritis was diminished after accounting for obesity, possibly indicating that OSA and obesity synergistically contribute to chronic gastritis.

Inflammatory bowel diseases (IBD), including Crohn’s disease and ulcerative colitis, represent chronic inflammatory condition of the gastrointestinal tract. The pathogenesis of intestinal lesions in IBD remains elusive. The bidirectional interactions between sleep disorders and IBD have recently received considerable attention (57, 58). OSA causes intermittent hypoxia, which results in periodic reductions in the oxygen partial pressure gradient within the lumen, promoting intestinal dysbiosis and inflammation (59). Disruption of the circadian rhythm can impair normal intestinal immune system activity, leading to an increased release of inflammatory factors and exacerbating intestinal inflammation (60–62). A retrospective study using US-wide diagnostic coding data independently linked IBD to a higher prevalence of OSA (63), consistent with the findings of this MR analysis. The potential causal relationship between genetically predicted OSA and ulcerative colitis identified in this study may provide new perspectives to explore the pathogenesis and treatment of IBD.

Moreover, we observed a positive link between OSA and gallstones, including calculi in the gallbladder and bile duct. This outcome is consistent with the finding from a cross-sectional study conducted by Chen et al. on 3,827 patients (7). OSA may cause gallstones by impeding gallbladder contraction through stimulating sympathetic nerves and enhancing bile supersaturation through inflammation associated with chronic intermittent hypoxia (64, 65). A previous Mendelian analysis showed that 1−standard deviation increase in the body mass index resulted 1.631 times increased odds of developing gallstones (66). However, the current MVMR analysis found that the causal relationship between OSA and gallbladder calculi was no longer significant after considering obesity; the causal relationship with bile duct calculi remained. This result suggests that OSA may affect gallbladder calculi, but not an independent risk factor.

Recent research has revealed a connection between OSA and the initiation and progression of NAFLD (67), indicating that patients with OSA face poor prognoses and are susceptible to other liver conditions (68, 69). However, MR results did not reveal a significant causal association between genetic predisposition to OSA and the development of NAFLD or cirrhosis. In addition, separate epidemiological studies by Nieto et al. (70) and Marshall et al. (71) reported significantly higher cancer mortality rates among patients with sleep apnea. However, Kendzerska et al. (72) concluded that OSA severity does not increase cancer incidence. Our study found no correlations between genetically predicted OSA and gastrointestinal cancers, including esophageal, gastric, or colorectal cancers. These findings suggest that the observed epidemiological relationships may stem from shared genetic components or unmeasured confounding factors.

This study had several advantages. First, this study is the first to comprehensively assess the cause-and-effect relationship underlying OSA with major GDs. We utilized two-sample MR based on SNPs from large sample populations (33,423 cases and 307,648 controls for OSA; an average of 9,530 cases and 299,113 controls for each GD); the sample sizes for exposure and outcomes did not overlap. Second, multiple MR statistical methods and multivariate MR analyses were applied to minimize confounding bias and guarantee the reliability of the findings, including adjustments for obesity, T2D, smoking, and alcohol consumption.

This study had limitations. First, the summary-level data from the GWASs included trans-ancestry populations among the source populations, as shown in **Table 1**. This inclusion could bias results because of the possibility of population mixture confounding. Second, MVMR analysis was performed for common risk factors for OSA and GDs, but other potential confounders may have a potential impact on the independence of causal association. Third, completely excluding the possibility that the SNPs linked to GERD affect OSA via additional causative pathways (i.e., horizontal pleiotropy) was challenging.

## 5 Conclusion

This MR analysis provides compelling evidence of an independent causal relationship between genetically predicted OSA and an elevated risk of multiple inflammation-related GDs. In summary, this study provides strong evidence that OSA and GERD are independent risk factors for each other. In addition, a novel independent causal association was found between genetically predicted OSA and gastroduodenal ulcer. The increased risk of chronic gastritis, ulcerative colitis, and gallstones (including calculus in the gallbladder and bile duct) may also be correlated directly with OSA. Understanding the biological pathways and mechanisms underlying these correlations will aid physicians and researchers in developing preventive and therapeutic strategies for patients with OSA presenting with gastrointestinal symptoms.

## Data availability statement

The original contributions presented in the study are included in the article/**Supplementary Material**. Further inquiries can be directed to the corresponding authors.

## Author contributions

WY: Conceptualization, Data curation, Formal analysis, Methodology, Software, Visualization, Writing – original draft. JZ: Data curation, Investigation, Validation, Writing – original draft. MJ: Conceptualization, Supervision, Validation, Writing – review & editing. YK: Data curation, Formal analysis, Writing – original draft. HQ: Data curation, Visualization, Writing – original draft. YQ: Data curation, Formal analysis, Writing – original draft. SW: Conceptualization, Investigation, Supervision, Validation, Writing – review & editing. JT: Conceptualization, Funding acquisition, Writing – review & editing.

## Supplementary material

Supplementary Figure 1 Scatter plot of the effect of the potential causal relationship between OSA and GDs. Figures **(A–G)** show the MR results in the significant causal relationship between OSA and GDs. The slope equals the b-value calculated using the five methods, representing the causal effect. The positive slope indicates that exposure is a risk factor, whereas a negative slope is the opposite. OSA, Obstructive sleep apnea; GDs, gastrointestinal diseases; MR, Mendelian randomization; SNP, single nucleotide polymorphisms; IVs, instrumental variables.

Supplementary Figure 2 Funnel plot of the effect of the potential causal relationship between OSA and GDs. Figures **(A–G)** show the distribution of IVs in the significant causal relationship between OSA and GDs, respectively. Red boxes indicate potential outliers that were eliminated from the analysis. OSA, Obstructive sleep apnea; GDs, gastrointestinal diseases; MR, Mendelian randomization; SNP, single nucleotide polymorphisms; IVs, instrumental variables; SE, standard error.

Supplementary Figure 3 MR leave-one-out sensitivity analysis for ‘Gastroesophageal reflux disease’ on ‘OSA’. MR, Mendelian randomization; OSA, Obstructive sleep apnea.

Supplementary Figure 4 MR leave-one-out sensitivity analysis for ‘OSA’ on ‘Gastroduodenal ulcer’. MR, Mendelian randomization; OSA, Obstructive sleep apnea.

Supplementary Figure 5 MR leave-one-out sensitivity analysis for ‘OSA’ on ‘Chronic gastritis’. MR, Mendelian randomization; OSA, Obstructive sleep apnea.

Supplementary Figure 6 MR leave-one-out sensitivity analysis for ‘OSA’ on ‘Ulcerative colitis’. MR, Mendelian randomization; OSA, Obstructive sleep apnea.

Supplementary Figure 7 MR leave-one-out sensitivity analysis for ‘OSA’ on ‘Calculus of gallbladder’. MR, Mendelian randomization; OSA, Obstructive sleep apnea.

Supplementary Figure 8 MR leave-one-out sensitivity analysis for ‘OSA’ on ‘Calculus of bile duct’. MR, Mendelian randomization; OSA, Obstructive sleep apnea.

Supplementary Figure 9 MR leave-one-out sensitivity analysis for ‘Gastroesophageal reflux disease’ on ‘OSA’. MR, Mendelian randomization; OSA, Obstructive sleep apnea.
