# Supplementary material for: Obstructive sleep apnea and 19 gastrointestinal diseases: a Mendelian randomization study
Source: Front Psychiatry. 2024 Jul 26;15:1256116. doi: 10.3389/fpsyt.2024.1256116 (PMC11310136; doi:10.3389/fpsyt.2024.1256116)

Supplementary Figure 1

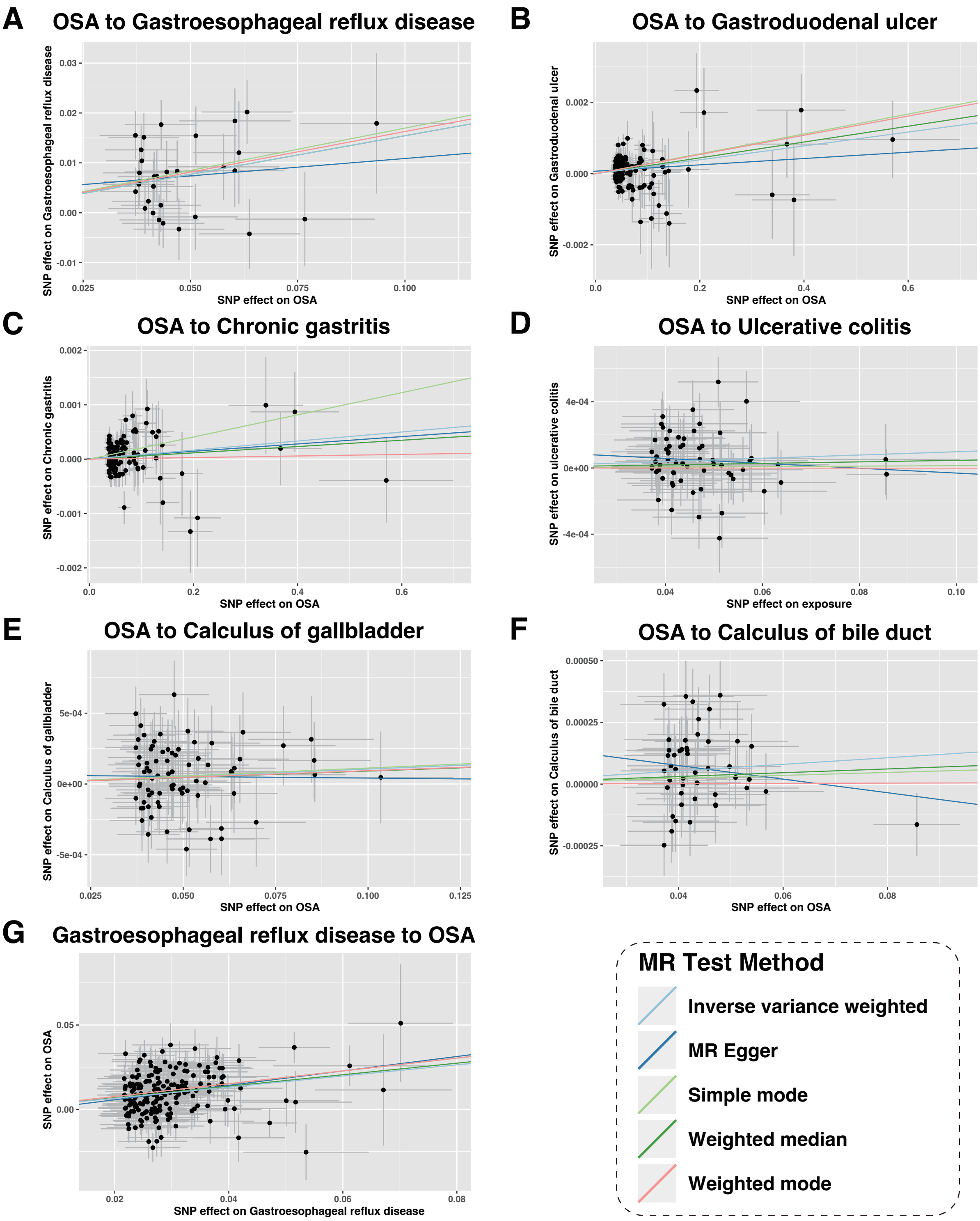

Supplementary Figure 2

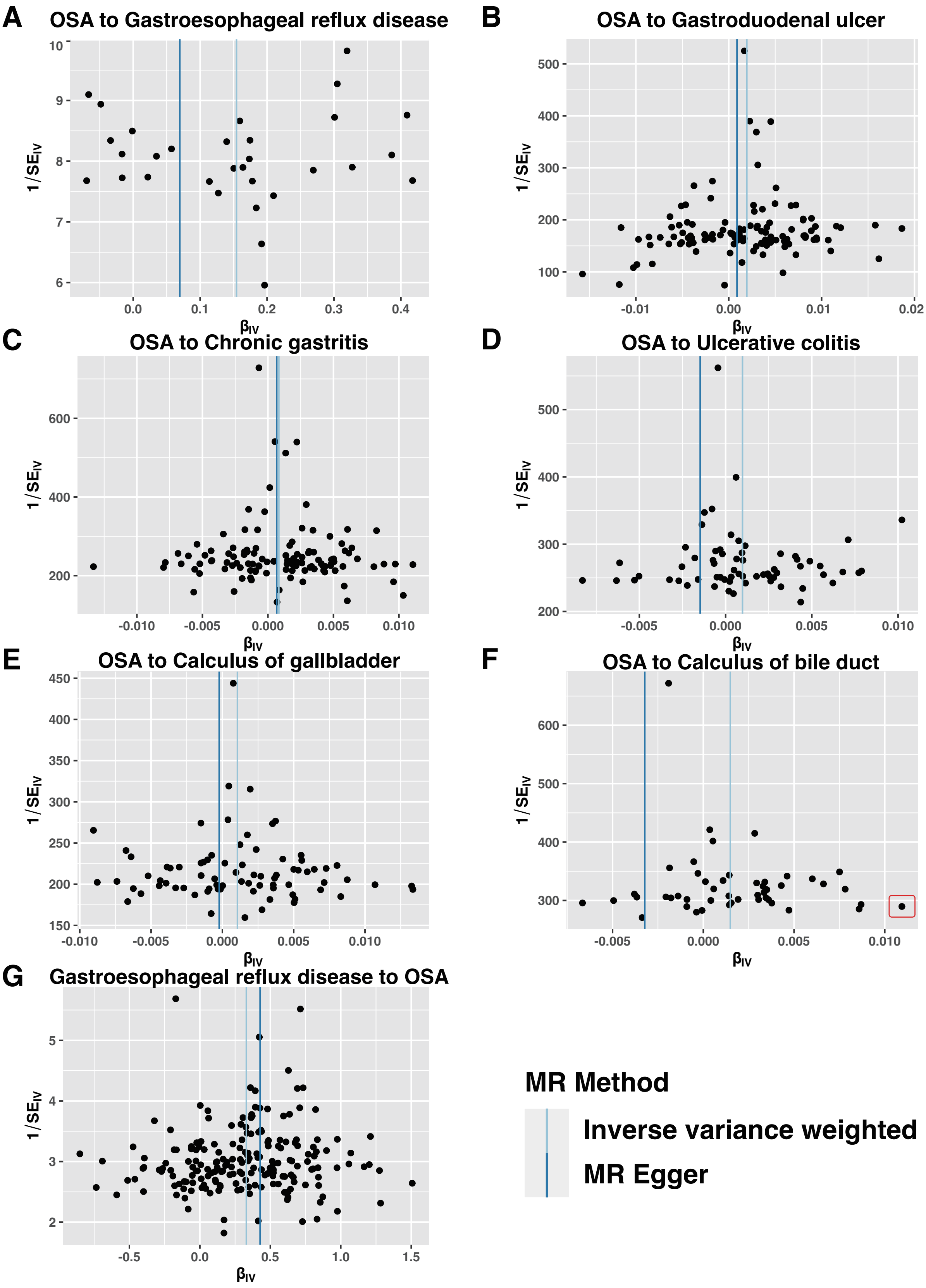

Supplementary Figure 3

MR leave-one-out sensitivity analysis for 'OSA' on 'Gastroesophageal reflux disease'

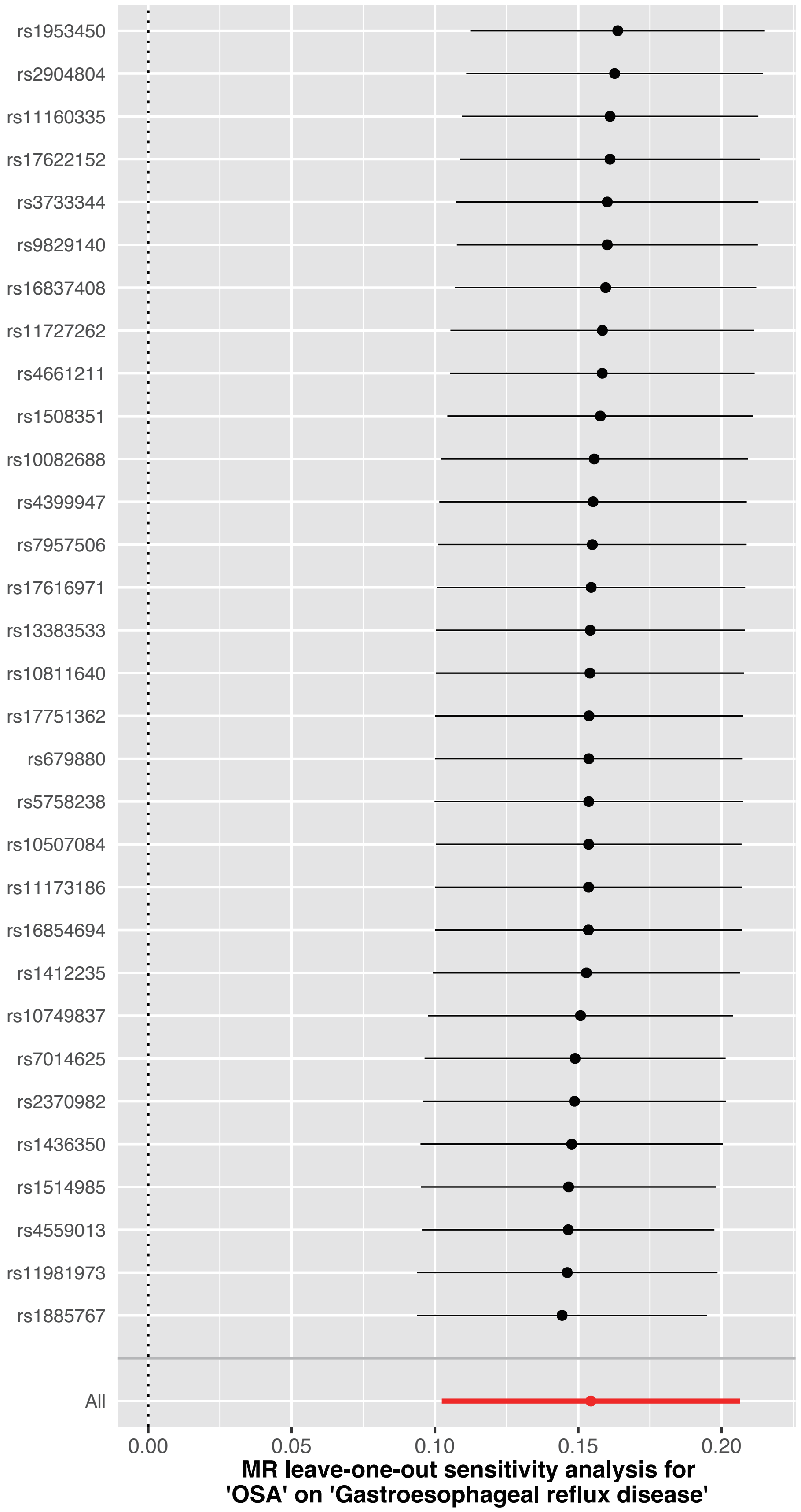

Supplementary Figure 4

MR leave-one-out sensitivity analysis for 'OSA' on 'Gastroduodenal ulcer'

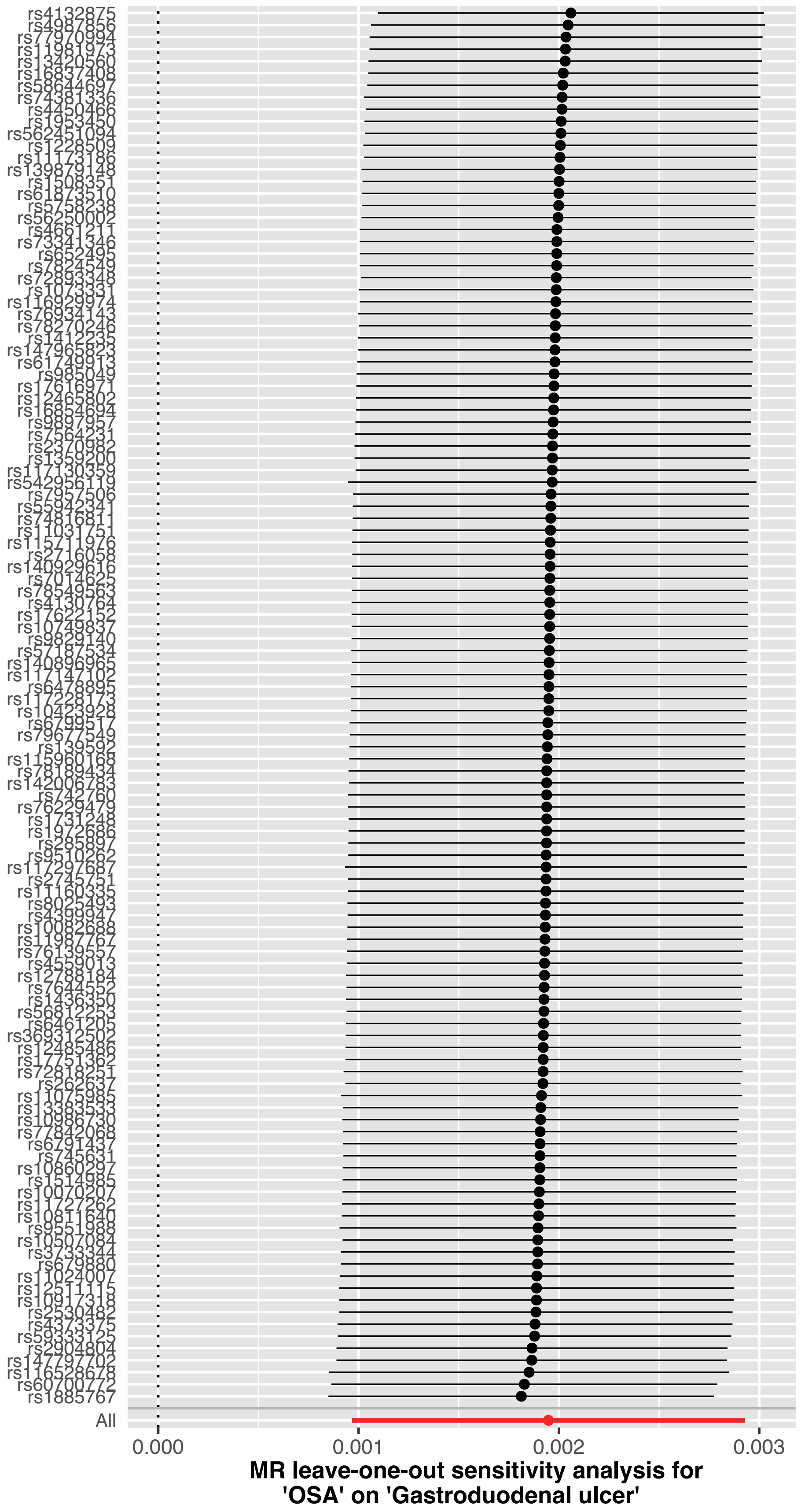

Supplementary Figure 5

MR leave-one-out sensitivity analysis for 'OSA' on 'Chronic gastritis'

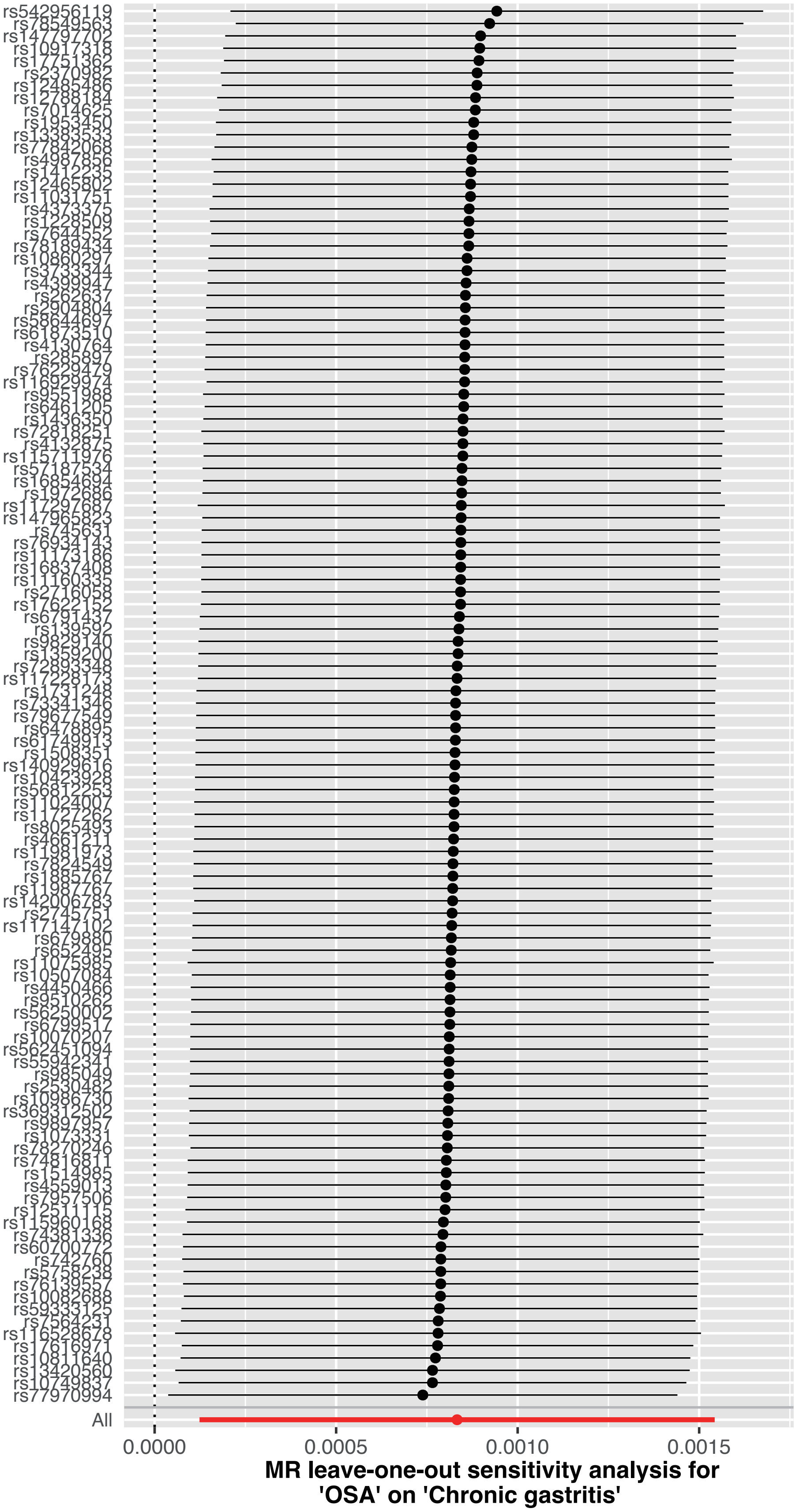

Supplementary Figure 6

MR leave-one-out sensitivity analysis for 'OSA' on 'Ulcerative colitis'

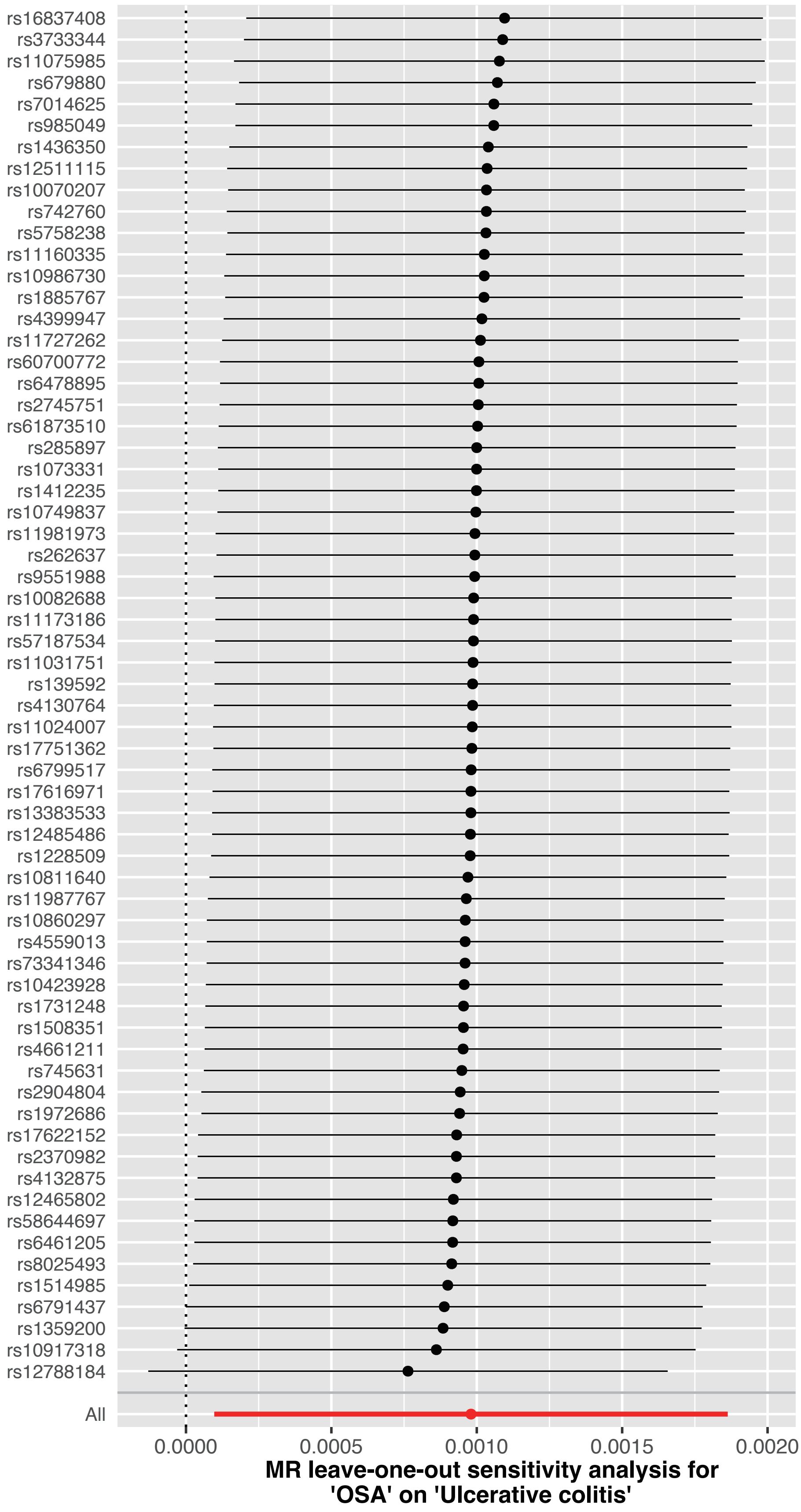

Supplementary Figure 7

MR leave-one-out sensitivity analysis for 'OSA' on 'Calculus of gallbladder'

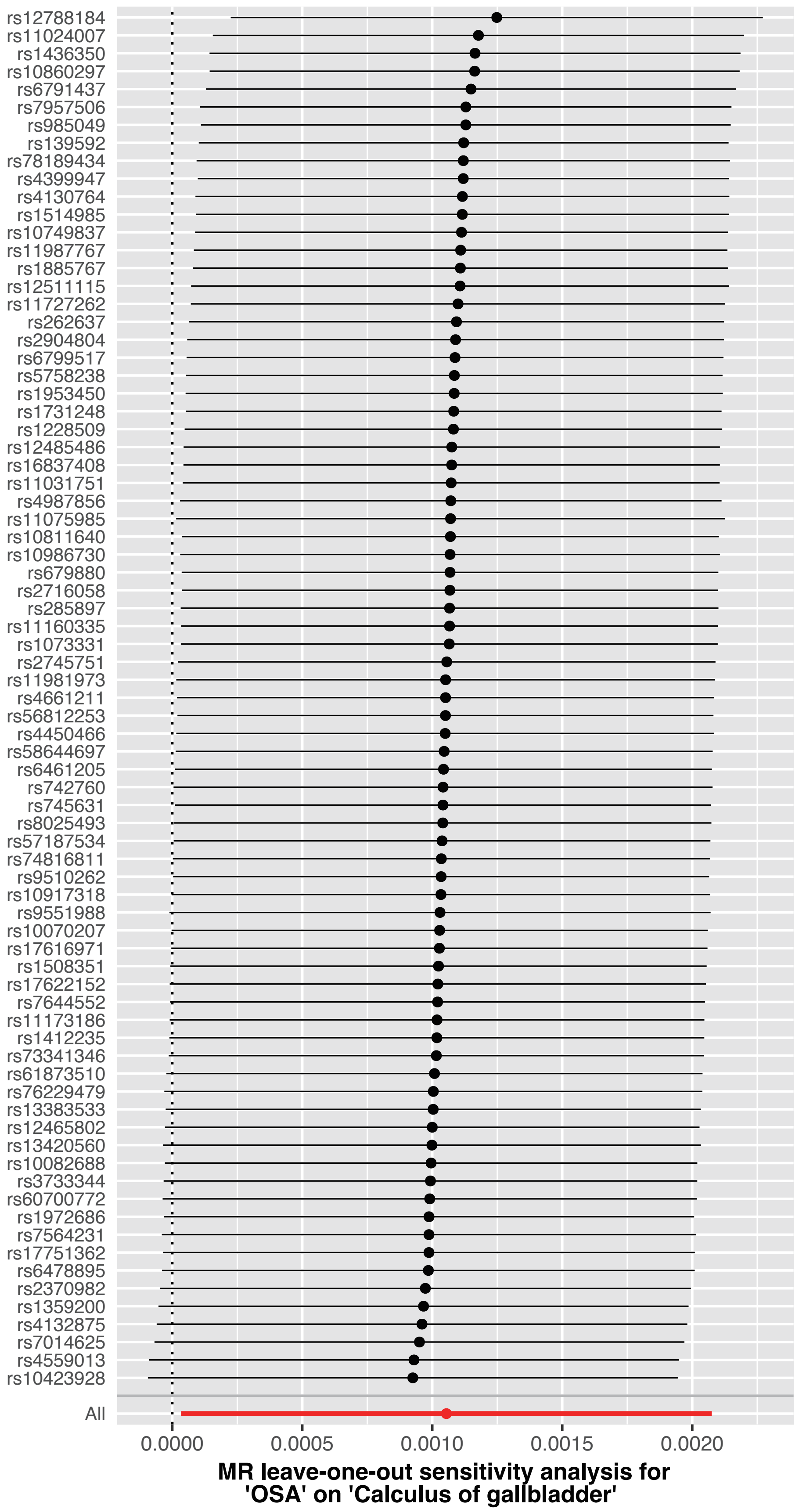

Supplementary Figure 8

MR leave-one-out sensitivity analysis for 'OSA' on 'Calculus of bile duct'

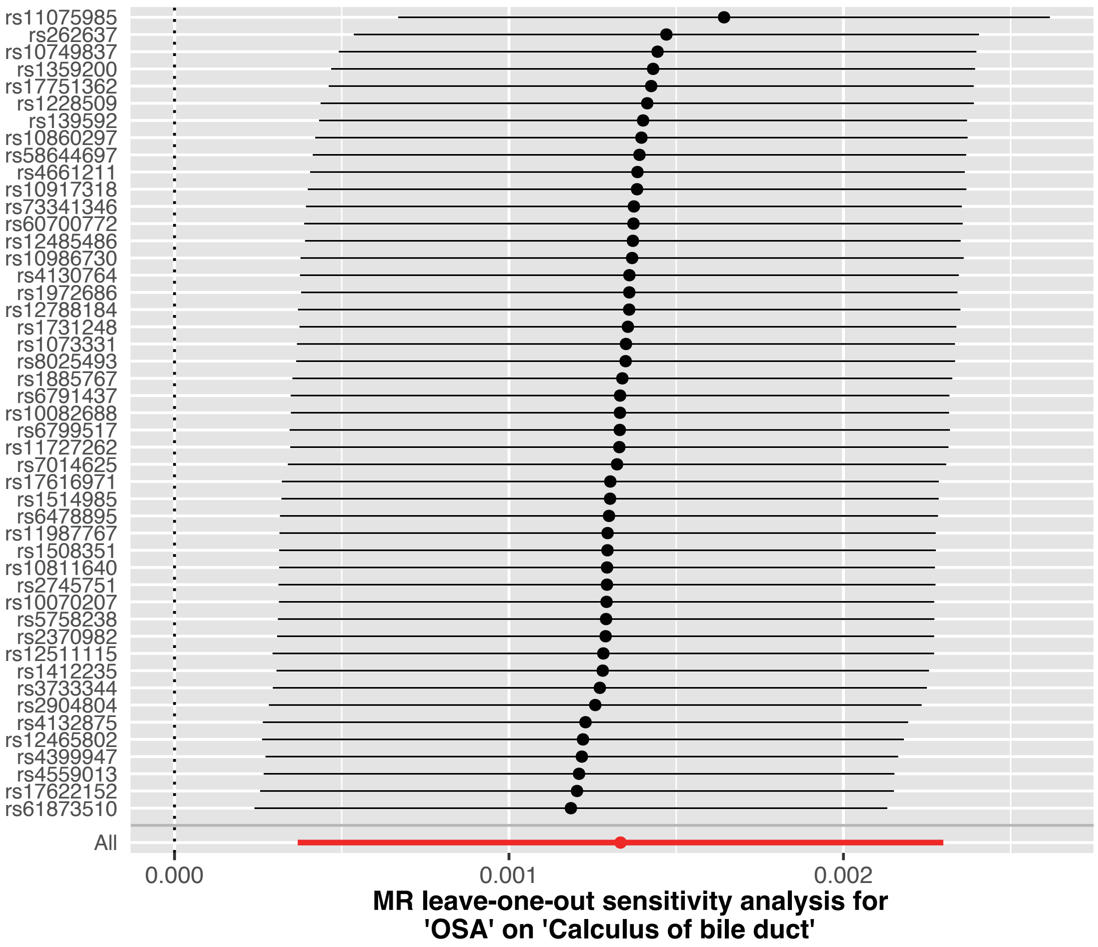

Supplementary Figure 9

MR leave-one-out sensitivity analysis for 'Gastroesophageal reflux disease' on 'OSA'

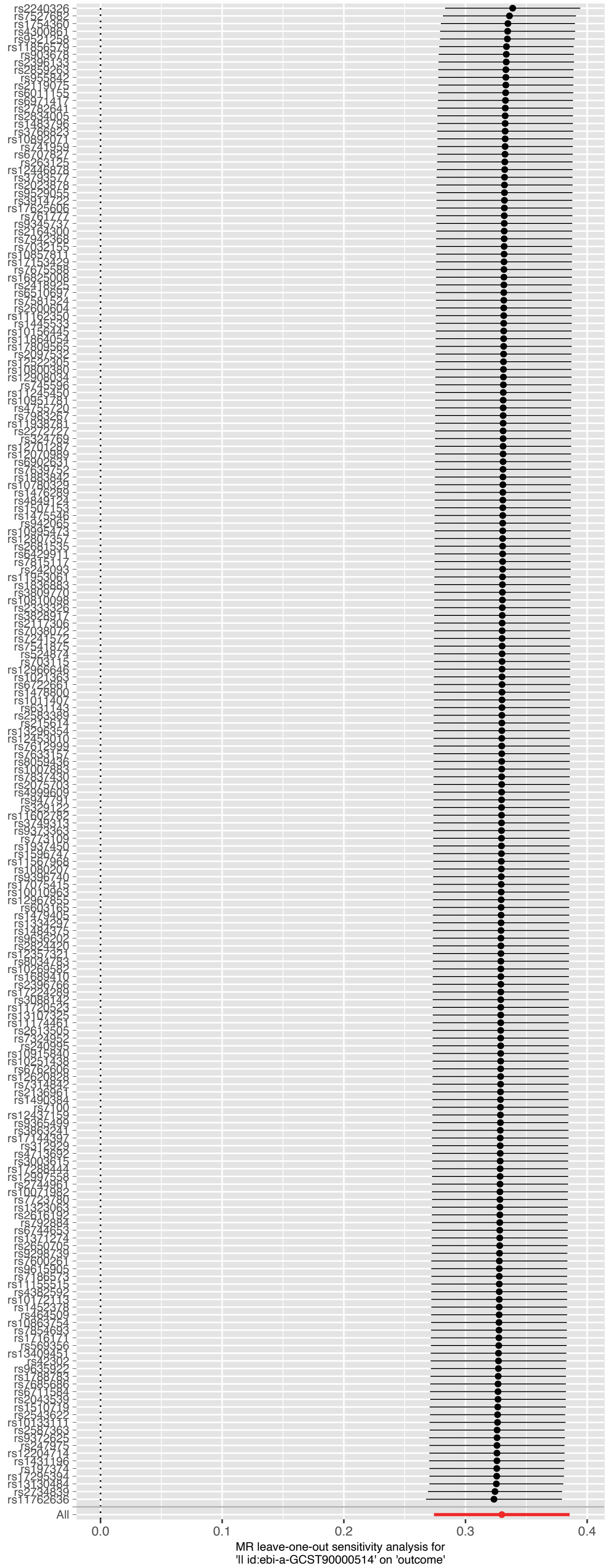

Supplement: Supplementary Figure 1 — Scatter plot of the effect of the potential causal relationship between OSA and GDs. Figures (A–G) show the MR results in the significant causal relationship between OSA and GDs. The slope equals the b-value calculated using the five methods, representing the causal effect. The positive slope indicates that exposure is a risk factor, whereas a negative slope is the opposite. OSA, Obstructive sleep apnea; GDs, gastrointestinal diseases; MR, Mendelian randomization; SNP, single nucleotide polymorphisms; IVs, instrumental variables. [file Image_1.pdf]
